# Forest Productivity Enhancement and Compensatory Growth: A Review and Synthesis

**DOI:** 10.3389/fpls.2020.575211

**Published:** 2020-09-29

**Authors:** Chao Li, Hugh Barclay, Bernard Roitberg, Robert Lalonde

**Affiliations:** ^1^Canadian Wood Fibre Centre, Canadian Forest Service, Edmonton, AB, Canada; ^2^Pacific Forestry Centre, Canadian Forest Service, Victoria, BC, Canada; ^3^Department of Biological Sciences, Simon Fraser University, Burnaby, BC, Canada; ^4^Department of Biology, University of British Columbia-Okanagan, Kelowna, BC, Canada

**Keywords:** forest growth model, overcompensation, partial harvest, plant response manipulation, wood supply

## Abstract

This review and synthesis article attempts to integrate observations from forestry to contemporary development in related biological research fields to explore the issue of forest productivity enhancement and its contributions in mitigating the wood supply shortage now facing the forest sector. Compensatory growth has been clearly demonstrated in the long-term precommercial thinning and fertilization trial near the Shawnigan Lake, British Columbia, Canada. This phenomenon appears similar to many observations from other biological fields. The concept of compensatory growth can be applied to forest productivity enhancement through overcompensation, by taking advantage of theories and methods developed in other compensatory growth research. Modeling technology provides an alternative approach in elucidating the mechanisms of overcompensation, which could reveal whether the Shawnigan Lake case could be generalized to other tree species and regions. A new mitigation strategy for dealing with issues related to wood supply shortage could be formed through searching for and creating conditions promoting overcompensation. A forest growth model that is state dependent could provide a way of investigating the effect of partial harvest on forest growth trajectories and stand dynamics. Results from such a study could provide cost-effective decision support tools to practitioners.

## Introduction

Forest productivity has historically been a central concern in forestry, due to its close relation to timber production (Leuschner, 1984; Avery and Burkhart, 1994; Wenger, 1984) and ecosystem services such as biodiversity and wildlife habitat protection (Jenkins and Schaap, 2018; Felton et al., 2020) and carbon storage (Canadian Council of Forest Ministers, 2000; Davis et al., 2001). This concern has become urgent recently due to increasing market demands and potential climate change impact (IPCC, 2007), and enhancing concern for environmental protection (FAO, 2016). As a result, accurate estimation of forest productivity appears crucial in decisions of sustainable forest management. Here we attempt to provide a systematic review on the subject and apply the concept of compensatory growth (CG) to explain the forest dynamics after experiencing a partial mortality caused by both anthropogenic and natural disturbances. This synthesis is aimed at offering a new approach of handling wood supply shortage related issues.

### Forest Productivity

The term forest productivity has been widely used in forestry-related literature (Crow et al., 2006; Keeling and Phillips, 2007; Zhang et al., 2012; Van Bogaert et al., 2015). However, this term could mean different things to different people. Two meanings can be identified: *cumulative increase* and *net annual increase* of forest volume.

Odum (1959) distinguishes between *primary productivity* (the rate at which energy is stored by photosynthetic and chemosynthetic activity of producer organisms, chiefly green plants) and *secondary productivity* (the rate at which the carbon stored by primary producers is assimilated by animals or decomposers). Primary productivity is further divided into *gross primary productivity*, “the total rate of photosynthesis including the organic matter used up in respiration during the measurement period,” and *net primary productivity* (NPP), “the rate of storage of organic matter in plant tissues in excess of the respiratory utilization by the plants during the period of measurement.” In the context of a forest, NPP includes not only the biomass in trees, but also that in herbs and shrubs, although the biomass of herbs and shrubs is usually negligible compared with that of trees. These definitions have become standard in the ecological literature. Thus, the net productivity of trees (the items of interest to most foresters) usually closely approximate the NPP. The term “productivity” is noteworthy because it is a rate and involves acquisition of photosynthate per unit time.

From a forestry perspective, forest productivity is often defined as the standing forest volume at a given time *t*, *V_t_*, which is the cumulative increase of stand volume since the stand was initiated (at *t*=*t*_0_). It is referred to as yield in studies of forest growth and yield (Avery and Burkhart, 1994; Weetman and Mitchell, 2013). Typically, the term productivity is used to account for the accumulation of aboveground stem wood in standing trees, although it may also include below-ground accumulation. *V_t_* will generally increase with stand age and generally a plot of volume over time has a sigmoid shape that asymptotes at the site’s carrying capacity. Forest productivity is generally the basis for evaluating current forest inventory, planning bio-economy and sustainable forest management, and assessing current and future wood supply. This cumulative increase definition will be used in the current study. For carbon related studies, this definition of forest productivity is mainly concerned with carbon storage (Kurz et al., 1992; Kurz et al., 2009).

Research on forest productivity can be categorized in two ways. Most efforts have been focused on how to improve evaluation methods and accuracy of current forest productivity for a given site or forest (e.g., Martin, 1991; Huang et al., 2001). These efforts were aimed at deepening the understanding of the dynamics of ecosystems and increasing the potential of biomass production, which would improve regional carbon budgets. Such studies may also contribute to the evaluation of the effects of climate change (e.g., Biosvenue and Running, 2006). By contrast, other studies are devoted to exploring whether current forest productivity could be enhanced under normal conditions, and if so how and to what extent the enhancement could be implemented (e.g., Norby et al., 2010). Such studies enhance predictions as to future forest productivity under various conditions, including silvicultural operations.

Enhancing forest productivity will help meet the increasing wood utilization demands, especially when more frequent natural disturbances under a wide range of climate change scenarios could increase wood supply shortages (Li et al., 2000; Kirilenko and Sedjo, 2007).

Forest productivity enhancement can be viewed from two different approaches: *proactive* vs. *passive*. From a proactive perspective, the research focus is on the conditions that promote forest productivity enhancement. Here, one might view enhancement of forest productivity as a possible natural solution to resolve the issues related to wood supply shortage, because increased forest productivity would provide more wood volume being harvested for forest products such as lumber and pulp. The challenges involved with this approach include both scientific and operational investigations. From a biological and ecological view, the questions focus on what determines forest productivity, and whether and how it could be enhanced to satisfy increasing utilization demands, as well as ecosystem carbon dynamics and health and integrity as a whole. From an operational view, however, one would expect to see practical examples of forest productivity enhanced after some conditions have been applied such as silvicultural treatments. The stand volumes of the sites treated by precommercial thinning (PCT) exceeded those of control sites displayed in the long-term PCT effect studies of balsam fir in the Green River, New Brunswick (Pitt and Lanteigne, 2008) and Douglas-fir in the Shawnigan Lake trial in British Columbia (Li et al., 2018), which are examples of such treatments.

From a forest company’s operational perspective, however, both the proactive approach described above and the passive approach must be taken into account. The passive approach answers the question of what to do to make the best use of current forest productivity. This approach includes full, multiple, and optimal utilization of current forest productivity through matching the right fiber attributes with the right products, in the right place at the right market time (e.g., Li, 2009), and optimization technology in the operations research could play an important role. The passive approach also involves the direct application of the basic principles of forest economics. For example, Brodie and Tedder (1982) provided an example in considering the effect of regeneration delay on harvest scheduling for both surplus and deficit forest inventory. It can also include the design, analysis, and higher-level plans such as the application of global forest product models (e.g., Buongiorno et al., 2003). This approach needs to also take into account, the risk of natural disturbances, including fires, pests, windstorms, and extreme weather conditions (e.g., Li et al., 2000; Schelhaas et al., 2003; Keane et al., 2004; Seidl et al., 2011; Gustafson and Sturtevant, 2013; Kretchun et al., 2014; Gustafson et al., 2017; Navarro et al., 2018; Díaz-Yáñez et al., 2019; Lavoie et al., 2019), and the effects of climate change (Martin-Benito et al. 2008; He et al., 2017; Montoro Girona et al., 2019; Gustafson et al., 2020). In the current study, more attention will be paid to the proactive approach, i.e., directly working from biological and ecological enhancements, and we will leave the passive approach to others.

### Limits for Forest Productivity

Forest productivity is mainly limited by factors such as resource availability to the trees on site, and physical conditions at a site (Barnes et al., 1998; Odum and Barrett, 2005; Martin-Benito et al. 2008). These resources may include light, space, soil nutrients, and water, etc. Physical conditions may include elevation, topography such as slope and aspect, climate and weather variables and their dynamic patterns over time.

The theoretical expectation of resource-dependent forest growth can be traced back to the famous Clementsian forest succession concept of “forest ecosystem change occurring over a range of decades to centuries with resulting change in composition structure, and biomass of vegetation” (Barnes et al., 1998 p. 41). Forest succession has been stated as a “universal law” that “all bare places give rise to new communities except those which present the most extreme conditions of water, temperature, light, or soil” (Clements, 1916). Its significance persists and was described by Eugene Odum (1969) as critical for human society. Despite different viewpoints, critical reviews, and new perspectives, the basic principles of forest succession theory remain unchanged in three ways: (1) succession is an orderly process that is reasonably directional and therefore predictable; (2) succession results from modification of the physical environment by the community, i.e., “community controlled”; and (3) succession will culminate in a stabilized (climax, mature) ecosystem with homeostatic properties (McIntosh, 1981). From a perspective of plant population ecology, the biomass of a population will increase with time or stand age and gradually and eventually reach a plateau or mature (climax) state.

The conventional theory of forest succession predicts the growth of a forest over time up to a level of dynamic equilibrium represented by a climax (mature) community. This general pattern, in a graphical form, can be characterized as a sigmoid shape with volume over time increasing until an asymptotic status is reached wherein the level of the plateau will be determined by the site carrying capacity, which represents the maximal capability of supporting and maintaining biomass at the site. Consequently, forest growth models at the stand level also generally have such a form. However, increasing observations of age-related decline in forest productivity suggest that forest growth patterns might not always be congruent with the predictions from forest succession theory, at the community level (e.g., Ryan et al., 1997; Binkley et al., 2002; Berger et al., 2004; Zaehle et al., 2009; Kuuluvainen and Gauthier, 2018).

Similar theories are also reflected in the study of the relationship between forest productivity and stand density, as summarized in Baskerville (1965) as follows:

“There are two principle theories dealing with the correlation of forest production to stand density. The one put forward by E. Assmann states that growth per unit area increases with increased stocking until optimum production is reached at some definable density. Beyond this point, production decreases. Assmann used basal area expressed as a percent of the basal area of fully stocked stand as his measure of density. His hypothesis was developed largely from an analysis of classical European yield tables and from observation of European thinning experiments. Assmann held that optimum production occurred within a very narrow range of densities and only on exceptionally good sites would the curve be broad, indicating roughly equivalent production across a wide range of densities.

The second general hypothesis is that put forward by C.M. Möller. He postulated that production increases with increased stocking up to the point of full occupancy of the site densities hence production should be equivalent provided the balance of non­photosynthetic area to photosynthetic area did not exceed critical limits. Respiration would become limiting only in very high density stands where the surface area of boles and branches (the non-photosynthetic respiring area) increases greatly. Möller tested his hypothesis using Danish thinning practice in Norway spruce and beech and found it was upheld.

The two hypotheses are seen to differ only in respect to the range of densities across which production is optimum. Of critical importance therefore is the parameter of density. There is a need for a measure of stocking which is related to full occupancy (carrying capacity in the sense of population dynamics) in absolute terms. One would expect that for a given soil there must be a maximum rate at which nutrients and water can be made available to plants and that rate determines the amount and nature of roots occupying that soil. Similarly the maximum amount of foliage a given species, or combination of species, can effectively display to the sun determines the upper limit of intercepted, and hence effective radiation. The interaction of these maxima would produce the greatest dry-matter production; all other combinations would produce lesser amounts. It would be convenient to express this measure of stocking in terms of a common stand parameter such as basal area per acre.”

Resource-dependent forest growth is also closely related to the concept of carrying capacity, which has been used widely in theoretical biology and population ecology for characterizing the maximal capacity of biomass that can be supported and maintained for a given site (Martire et al., 2015). Hui (2006) summarized that the carrying capacity of a biological species in an environment is the maximum population size for that species that the environment can sustain indefinitely, given the food, habitat, water, and other necessities available in the environment. In population biology, carrying capacity is defined as the environment’s maximal load. It is different from the concept of population equilibrium. Its effect on population dynamics may be approximated in a logistic model, although this simplification ignores the possibility of overshoot, which real systems can exhibit. In mathematical form, the carrying capacity usually has an abbreviation of *K*, and it is usually called *K* value. Note the *K* value for any site likely varies over time.

In nature, the pattern of forest growth over time could be influenced by a number of factors such as site index and species (King, 1966; Means and Helm, 1985; Martin, 1991). In contrast with *K*, a quantitative parameter, the site index includes both quantitative and qualitative parameters. It is defined as the tree top height at the age of 50 years, and can be seen as a combined indicator from various nutrients, water, and site physical conditions. Site index also represents resource availability at the site when other physical site conditions are fixed. Due to differing growth and maturation requirements, different tree species could also respond to the site conditions in different manners. As a result, forest growth equations have demonstrated diverse forms in different geographical locations for different tree species. For this reason, forest growth models are site-specific and species-specific.

The concept of site index has been critical in the development of forest growth and yield models (Martin, 1991; Huang et al., 2001). The site index is a measurement commonly used by foresters to describe the productivity of a site, usually defined as dominant tree height at 50 years old (King, 1966). One example of its utility comes from the development of the TASS model in British Columbia, Canada, which stands for Tree and Stand Simulator (Mitchell, 1975). All the simulations are based on the estimated site index, and tree growth is proportional to the crown size. In other words, site index determines the upper limit of tree growth—maximized crown size—and size and shape of tree stems are determined by the realization of the maximized crown size.

### Thinning Induced Compensatory Growth and Overcompensation

It has been widely observed that tree growth, in terms of diameter and total height, in PCT trials can result in increased growth rates relative to that from untreated sites in the short term (e.g., Bose et al., 2018). This is known as released growth of the remaining crop trees (Miller et al., 2007). It is easy to see that PCT reduces stand density and results in trees with larger diameters than those from untreated sites. This outcome is so common that it has been routinely applied in forestry practices to obtain tree forms that are most desirable for later processing into lumber products.

Intuitively, if this increased growth rate persists in the long term, any volume initially lost in PCT operations will be replaced over time and eventually the total stand volume will equal or even exceed stand volumes produced in untreated sites (Montoro Girona et al., 2017). Individual tree-based models (e.g., Korzukhina and Ter-Mikaelian, 1995; Battaglia and Sands, 1998; Ancelina et al., 2004) could be an interesting tool to understand this inference. This phenomenon, however, is not often reported in the literature due to the short period of observation remaining after PCT as mandated by present guidelines. For example guidelines mandate a post-PCT observation period of only 10 years for Douglas-fir (e.g., Reukema, 1975). Nevertheless, this phenomenon has been described in some long-term PCT trials such as one on balsam fir in Green River, New Brunswick, Canada, in which the stand volumes in treatment sites were about 15% higher than those from untreated sites, 42 years after the initial treatments (Pitt and Lanteigne, 2008). However, the mechanisms underlying these kinds of results are a matter of some debate. Due to its one-time measurement (i.e., measurement was made on trees at final felling), many questions could not be properly answered (re-measurement is no longer possible), and many researchers concluded that the results were simply an anomaly. Nevertheless, with the discovery from a data analysis of 40-year PCT and fertilization trial in Shawnigan Lake, British Columbia, Canada, which contained multiple measurements of the sites, it has become clear that the released growth of surviving trees can persist into the long term indeed and result in the stand volumes in treated sites that equal and exceed those from untreated sites (please see *Shawnigan Lake PCT and Fertilization Trial Overview* for a detailed overview of the case analysis). A growth response pattern to PCT is similar to the CG observed in other biological research fields; consequently, the framework of CG was used to explain the observation (Li et al., 2018). CG is a special case of growth response specifically for growth responses after thinning operations, and overcompensation has been used to describe the state when stand volumes in treated sites exceed the ones from untreated sites. CG could be extended to include shelterwood treatments (Bose et al., 2014; Pamerleau-Couture et al., 2015; Montoro Girona et al., 2016, Montoro Girona et al., 2018; Bose et al., 2020) and windstorm (Coates, 1997; Ruel et al., 2003; Montoro Girona et al., 2019).

The observed overcompensation from the Shawnigan Lake trial appears undisputable and is of great interest in terms of deepened understanding of the roles of thinning operations and search for conditions that promote forest productivity enhancement. If the role of PCT is merely for stand density management, forest productivity resulting from careful design of spacing during plantation and from PCT operations should be more or less the same. In other words, perfect design of spacing during planting should produce the same effect as without spacing in PCT operations. Harvested wood in PCT operations has no significant commercial value as lumber or generally any other forestry commodity. However, jack pine tree research (Tong and Zhang, 2005) has shown that the effects of initial spacing and the effects of subsequent PCT are probably not the same. Apparently, best results in terms of tree growth and stem quality can be expected from a narrow initial spacing during planting, followed by a later PCT.

Overcompensation is equivalent to enhanced forest productivity. In other words, if the mechanisms responsible for overcompensation can be understood, forest productivity could be enhanced through creating the right conditions, and forestry practices could benefit from applying operations that create such conditions. To reach this goal, the following questions need to be answered:

- What is/are the mechanism(s) behind CG and overcompensation?
- What factors trigger or determine the CG and overcompensation?
- Does CG aid in productive and sustainable outcomes, and can it persist over time?
- If PCT can serve as a stimulus to the remaining trees, what sorts of optimal thinning operations are needed to obtain maximized overcompensation?
- And eventually, is it possible to predict CG and overcompensation?

### Objectives of This Review and Synthesis

The objectives of this review and synthesis article are twofold: to provide a conceptual framework for analyzing CG and to estimate CG contributions to forest productivity enhancement. *Forest Productivity Prediction* provides an overview of different modeling approaches and examples of characterizing forest growth. *Role of Compensatory Growth* presents a general overview of CG from different perspectives, a definition and numerical examples of CG, a brief description of CG observed from the Shawnigan Lake PCT and fertilization trial, and possible explanations of general CG from related research fields. *Possible Generalization of Overcompensation to Other Species and Geographical Regions* provides a summary of possible approaches to generalize overcompensation to other tree species and geographical regions, and the pros and cons associated with them. This article will conclude with some research recommendations.

## Forest Productivity Prediction

Forest productivity at any given site varies over time, subject to normal birth, growth, and death processes of trees (Botkin et al., 1972; Botkin, 1993; Bugmann, 2001), as well as from different natural and anthropogenic disturbance regimes including fire (Prentice et al., 1993; Li and Apps, 1995; Keane et al., 1996; Li and Apps, 1996; Li et al., 1997; Li, 2000; Li et al., 2005; Barclay et al., 2006; Li and Barclay, 2008), pest (Safranyik et al., 1999; Safranyik et al., 2001; Li et al., 2005; Hennigar et al., 2008; Li et al., 2008), climate (Allen and Breshears, 1998; Li et al., 2000) and weather such as windstorm (Ruel et al., 2003), partial harvest and fertilization (Aber et al., 1978; Aber et al., 1982), etc. Substantial initiatives and different modeling approaches have been used to predict the dynamics of forest productivity, and these efforts could be categorized into age-dependent, resource-dependent, and state-dependent forest growth models that depend on both internal and external states of trees.

### Age-Dependent Forest Growth Models

Conventional age-dependent growth models are mainly empirical, data-based, statistical models commonly used for forest inventory evaluation, yield table generation, future wood supply forecast, and sustainable forest management (including harvest) planning. These models are site and species specific, and intensive field data collection, such as permanent sampling plot (PSP) and temporal sampling plot (TSP), are required to estimate parameters and improve the accuracy of the model predictions. These models predict the temporal dynamics of forest productivity and generally have a sigmoid shape wherein their saturation levels are determined by the carrying capacity of the site. These models reflect the natural courses of different stand types and provide tools for suitably estimating the productivity of natural stands.

The age-dependent forest growth models have the longest history of development and have the most mature form compared with other modeling approaches. The accuracy of model predictions has been improved over time through the enhancement of modeling systems. For example, the early growth and yield prediction system in Alberta was developed in 1984 from the phase III of the provincial AVI program (Alberta Forest Service, 1984; Huang, 1994). The system was relatively simple in equation form and provided an easy method of projecting current forest inventory into the future. It has been used in a spatially explicit model for landscape dynamics for reconstructing natural fire regimes (Li, 2000). The growth and yield prediction system (GYPSY) was developed in 2001; it provided a much more detailed and accurate description of forest growth and yield in the province for different tree species in different regions (Huang et al., 2001). The GYPSY model has been the standard growth and yield prediction system for Alberta and was further refined in 2009 (Huang et al., 2009). However, in the past two decades, increasing evidence suggested age-related decline that is inconsistent with the existing forest succession theory (e.g., Gower et al., 1996; Ryan et al., 1997; Berger et al., 2004). Some of the age-dependent forest growth models have incorporated this phenomenon (without specifying a particular mechanism), such as in Manitoba and Saskatchewan, Canada (Manitoba Conservation and Water Stewardship, 2013; Saskatchewan Ministry of Environment, 2017). These observations raised a question of their effect on the accounting of site productivity. Nevertheless, this topic is beyond the scope of the current review and synthesis.

Growth and yield prediction systems are usually region-specific and developed for difference provinces of Canada. In British Columbia, for example, The Ministry of Forests, Lands and Natural Resource Operations developed a tree level growth and yield model named TASS (Tree And Stand Simulator), which predicts the potential growth and yield of even-aged, single-species, managed stands for ten commercial tree species in British Columbia (Mitchell, 1975). A provincial system for predicting growth and yield of natural stands is also developed (Martin, 1991). These BC models are also used in Yukon for adjusted site levels. In Alberta, in addition to the GYSPY model, a Mixedwood Growth Model (MGM) is also developed (Bokalo et al., 2013). These Alberta models are also used in Northwest Territories. MGM and MGYPSY (adapted from GYPSY) are being used in Manitoba. In Ontario, Penner (2008) provided yield prediction for mixed species stands, and documented validation of empirical yield curves (Penner et al., 2008). In Quebec, a stand-level growth and yield model named NATURA-2009 is available for even-aged stands to predict the growth of merchantable trees (Pothier and Auger, 2011). For uneven-aged stands, a tree-list growth and yield model named Artemis-2009 is available (Fortin and Langevin, 2010). In New Brunswick, a stand-level growth and yield is forecasted using a tree list model named Open Stand Model (OSM), which is similar to the Forest Vegetation Simulator (FVS) model (Dixon, 2002), but calibrated for the Acadian Forest region using the provincial permanent sample plots.

Ultimately, an age-dependent forest growth modeling system can provide estimates of forest volumes for particular tree species at specific sites at any stand age. In other words, under ideal situations, age-specific forest volumes can be calculated once the species and site conditions are known.

### Resource-Dependent Forest Growth Models

Resource-dependent forest growth models are mainly useful in stand density management practices for determining how forest volumes of a stand can be altered by different stand densities. There are three main categories of these forest growth models: variable-density yield tables, stand density management diagrams, and process-based mechanistic models.

Variable-density yield tables are a conventional yield prediction method developed to account for the yield changes due to different stand densities. Such efforts can be traced back to 1940s for natural stands (e.g., Mulloy, 1944). Johnstone (1976) developed a technique to facilitate assessment of past stand development from single-examination-plot PSP data to construct the yield tables for essentially pure, natural, and unmanaged, even-aged lodgepole pine stands of Alberta. The effect of stand density on top height of trees was represented by the following equation:

(1) $$\begin{array}{l} \log_{10}TopHt=1.0688-0.00276672A+0.717922Log_{10}A\\ -0.0000371084Stems-0.132622Log_{10}Stems \end{array}$$

where *TopHt* is the top height defined as the arithmetic average height of the 100 largest trees per acre, *A* is the stump-height age measured at 1.0 foot above ground level, and *Stems* is the total number of living trees equal to or greater than 0.6 inch diameter at breast height (DBH) outside bark. Using this equation, the author generated several yield tables for stands ranging in age from 20 to 100 years, high- to low-productivity sites, stand densities of 500 to 2,000 stems per acre at age 70 years, and quadratic-mean diameters from 3.0 to 8.5 inches at the same age.

The stand density management diagram (SDMD) is a graphical tool for relating stand density, tree size, and stand yield. It is a graph of mean tree volume and stand density on which the maximum size-density relationship has been superimposed. The relationship is based on the concept of maximum size density as a general principle of plant population biology: in pure even-aged stands, the maximum mean tree size attainable for any density can be determined by a relationship known as the −3/2 power law:

(2) $$Vol=\alpha Density^{-3/2}$$

where *Vol* is mean stand volume, *Density* is stand density, and *α* is a constant.

A SDMD is constructed by plotting mean tree volume against stand density on a double logarithmic scale (Drew and Flewelling, 1979). Two lines, one showing the maximum size-density relationship and another representing the lower limit of the zone of imminent competition mortality are usually included in most SDMDs. Several isolines are also drawn for stand top heights (representing height growth as a measure of site productivity) and quadratic mean diameter of a stand (representing stand diameter at a given age is altered given changes in establishment density). A crown closure line is also drawn and represents the points at which the crowns of trees in stands of different densities start to interact. These isolines can be calculated through multiple simulations using age-dependent forest growth models. For example, Farnden (1996) was able to use simulation results of a TASS model to construct SDMDs for lodgepole pine, white spruce, and interior Douglas-fir of British Columbia, Canada.

Both variable-density yield tables and stand density management diagrams are obtained from summaries of multiple simulations using empirical data-based, age-dependent, forest growth models. In other words, they are essentially an extension or application of age-dependent forest growth models.

Resource availability is a limiting factor in tree growth, and trees will likely grow faster with increased resource availability. Intuitively, therefore, trees that remain after partial harvest or other stand density management procedures would have extra resource accessibility and hence increase their growth rates. Consequently, resource-dependent forest growth models should be able to demonstrate the increased growth, either in DBH or *TopHt*, in stand dynamics after partial harvest. Such increased growth rate will inevitably result in observed CG, probably under CG, and eventually fully compensate the volume loss from the partial harvest operations. Nevertheless, it might be difficult to demonstrate overcompensation because of tree growth ceiling due to resource availability on site.

Although average stand volume can be estimated from these resource-dependent forest growth models, the effect of uneven spatial distribution of resources on the tree growth is rarely taken into account, even though this might be important in explaining the variations displayed in observations. Mechanistic modeling approaches have thus been employed to develop the TASS model (Mitchell, 1975), which is the foundation of simulating forest growth of managed stands in British Columbia, Canada.

The TASS model is also resource-dependent by way of maximal tree growth that is limited by the maximum crown development. In the model design, tree growth is assumed to be a fraction of crown development and spatial variations of resources. The TASS model appeared sensitive and capable of simulating forest dynamics after frequent small disturbance events and subsequent CG (Figures 13–16, and 18 of Mitchell, 1975), though the CG terminology was not used in the document. The simulated basal area increment from a row thinning was less than that from a checkerboard thinning regime, and various intensities of deer browsing had little effect on the final yield; in short-term simulations, their results likely describe growth during the phase of undercompensation. Nevertheless, the phenomenon of overcompensation may still be a question of representation in the simulation results if the simulations had continued over the long term. For this reason, running long-term simulations using TASS to reproduce the Shawnigan Lake trial could serve as a test and examination.

The overcompensation phenomenon observed in the long-term PCT trial in the Green River case and the combined PCT and fertilization trial in the Shawnigan Lake case might be difficult to explain using resource-dependent forest growth models because available resources are assumed to be utilized in full by normal stands already. Therefore, we propose that a state-dependent forest growth modeling approach should be introduced.

### State-Dependent Forest Growth Models

Any given organism can be described by values associated with its various states, including size, age, energy reserves, etc. The logic behind employing such variables lies in the fact that growth, for most organisms, including trees, is state dependent. For example, large trees with extensive crowns can capture more light energy than smaller trees belonging to the same species (conspecifics), but at the expense of higher maintenance costs and greater risk of mortality from wind (see Loehle, 2016). The job of a forest ecologist is to determine the key states that drive tree growth and forest productivity and there is a huge number of possible states that could be measured. For example, in a simulation model based approach, Buckley and Roberts (2006) developed a tree growth model (Deducing Emergent Structure and Physiology Of Trees or DESPOT) with at least 15 state variables to determine optimal carbon allocation to control various physiological processes to maximize net carbon gain. All of these processes and state variables have been identified as potentially important drivers of growth, and the simulations allow for interactions among them in relation to net carbon gain. As noted by Buckley and Roberts, these sorts of models will likely not produce mathematically tractable solutions but can be used to inform predictive modeling. By contrast, plant response manipulation through optimal stimuli or thinning is the state-dependent analytical approach that produced insights and predictions. One drawback is that these models are limited to just a few state variables thus requiring researchers to identify key state variables.

At the forest level, a good way of identifying and grouping states is to choose bins of appropriate size and then assign trees to these bins, across the entire forest. For example, a biologically reasonable bin size for age state would be one year; however, that fine a scale might be computationally unmanageable for forests that are tens or hundreds of years old. For tree height, bin size might be in multiples of meters. In the end, the entire population can be described by a distribution of trees across bins. Note that within a particular state (e.g., size) all individuals in a given bin are assumed identical; however, as we describe more states (e.g., now include age) the number of permutations or dimensions increases. Even in an even-aged stand, size and growth rate can be highly variable (Boyden et al., 2008). Again, computational issues arise as the number of states and bins increases; this is particularly true for analytical approaches such dynamic programming, sometimes referred to as the curse of dimensionality (Mangel and Clark, 1988).

Whether using individual based simulations or more analytically tractable state-dependent models, it is possible to calculate forest productivity by summing up a given variable across all trees in a particular forest. The key here is that carbon accumulation is not a logical phenomenon but rather the outcome of well-understood physiological processes that vary across tree states in heterogeneous forests. Interactions among these state variables and their associated physiological processes may generate emergent properties that might not be intuitive from single parameter assessments. For example, these models should generate the −3/2 power relationship without being constrained to doing so. In fact a good test of these models is to compare their predictions with real world patterns.

## Role of Compensatory Growth

### Compensatory Growth

Forest growth response (GR) is the term widely used in forest science for describing measured variables such as DBH and *TopHt* from treated sites, compared with those from untreated control sites, or the forest growth trajectories including those after PCT and other silvicultural treatments such as fertilization (Montoro Girona et al., 2017; Oboite and Comeau, 2019; Puhlick et al., 2019). GR appears to be a straightforward description of how forests respond to any disturbance or stimulus, and can be applied at both the individual level and the population level of trees.

While GR covers a very broad range of growth trajectories under both normal and abnormal (either advantageous or disadvantageous) environmental conditions, CG focuses on the growth trajectories after certain disadvantageous conditions (Guillet and Bergström, 2006; Rea and Massicotte, 2010; Erbilgin et al., 2014). Therefore, CG is a special case of GR. Though both GR and CG can characterize forest response to PCT, CG might be more specific and appropriate than GR; regardless, the diverse observations (with and without overcompensation) need to be examined and possible mechanisms explained. In this regard, ideas from CG research such as compensation and overcompensation could be introduced, which can serve as the starting point for exploring possible mechanisms. There are two streams of CG research: psychological behaviors (in the broad sense, see a summary by Hoffman, 2020) and ecological/biological responses.

In the field of behavioral ecology, which differs from human-based psychology, an evolutionary perspective is usually used to consider trade-offs in terms of inclusive fitness from benefit-cost analysis (Mangel and Munch, 2005; Bourke, 2011; Ferriere and Michod, 2011; Davies et al., 2012). In other words, conscious reactions need not be used but rather the focus is on responses or reactions that are selected or favored by natural selection. As such, behavioral ecologists seek behavioral strategies that maximize an individual’s lifetime reproductive success (Kalbe et al., 2008). Such strategies could include changes in growth response as a function of resource availability (Hoi et al., 2013), competition (Segers and Taborsky, 2012), or abiotic conditions (Debecker and Stoks, 2019). Thus, behavioral ecologists might ask whether facultative overcompensation, under some conditions, could confer a selective advantage (i.e., the function) over inflexible conspecifics and how such responses could be generated (i.e., the mechanism).

The phenomenon of overcompensation was probably observed first in plants in the 1920s in crops such as cotton in which removal of early reproductive organs of flower buds and squares could stimulate further growth thus generating overcompensation in the final yield. As a result, Eaton (1931) proposed this could be a means of increasing cotton yield without influencing the quality of cotton fiber. The capacity of this CG and overcompensation appeared to persist until the end of the growing season. CG was also observed in animals such as rats (Osborne and Mendel, 1915; Osborne and Mendel, 1916; Jackson, 1937) in which accelerated growth was exhibited when food intake was restored following a period of shortage. Animals later became traditional main objects of behavioral ecology research probably because of their relatively fast reaction time and short lifespan, until McNaughton (1985) who found that grazing increased grassland aboveground productivity with optimal results at intermediate grazing intensities; he concluded that CG mechanisms might be the driving forces determining plant productivity.

Given the demonstration of CG across kingdoms, questions remained as to its evolutionary advantage. Plants have received more attention in recent years, especially with the publication of a special issue of “Beyond traits: integrating behavior into plant ecology and biology” (Cahill, 2015). The main differences between animals and plants are mobility and photosynthetic ability; nevertheless, there are many similarities such as those displayed in movement of root systems and competition for nutrients and light. We might expect there to be differences in the driving mechanisms between plants and animals, but the behavioral ecology focus on the function of CG remains.

Though many behavioral ecologists focus on individuals, it is possible to scale up to the population or community level. In that regard, forests have been reported to have different capabilities of growth compensation after disturbances, such as CG after PCT operations. However, difficulties in deciding whether or not to implement PCT in forestry practice requires a better understanding with mechanistic explanations and solutions to facilitate informed management decisions. Here, practitioners could benefit from studies of the possible mechanisms of compensation and overcompensation from behavioral ecology research. With these in mind, we now describe the current understanding of CG in behavioral ecology.

The most common mechanism of CG is resource-dependent protection and defense from disturbances. Essentially, it is a survival strategy of organisms under disadvantageous conditions (Mangel and Munch, 2005). An animal’s body size is usually an important characteristic influencing mating choice and reproductive success, thus increasing body size is often a surrogate for increasing life-time fitness, and is favored with the trade-off risk of being discovered and captured by predators. When disadvantageous conditions occur, nutrient intake could be reduced through lower quality or quantity of food sources with requisite decline in growth rate. When food sources are restored, animals would tend to increase nutrient intake and thus increase their growth rate to achieve normal growth. Many examples have been documented including in insects (Hoi et al., 2013; Debecker and Stoks, 2019), fish (Ali et al., 2003; Azodi et al., 2016; Gabriel et al., 2018), mammals (Bohman, 1955), birds (Wilson and Osbourn, 1960), grass (McNaughton, 1983), and livestock (Yambayamba and Price, 1991; Zubair and Leeson, 1996; Therkildsena et al., 2004). As a result, CG research is no longer a pure but also an applied science that benefits different industries including crop protection and pest management, herbivory and rangeland management, fishery management, and farm animal production. This subject goes beyond the scope of the current review, and thus can be summarized elsewhere.

**Figure 1:**
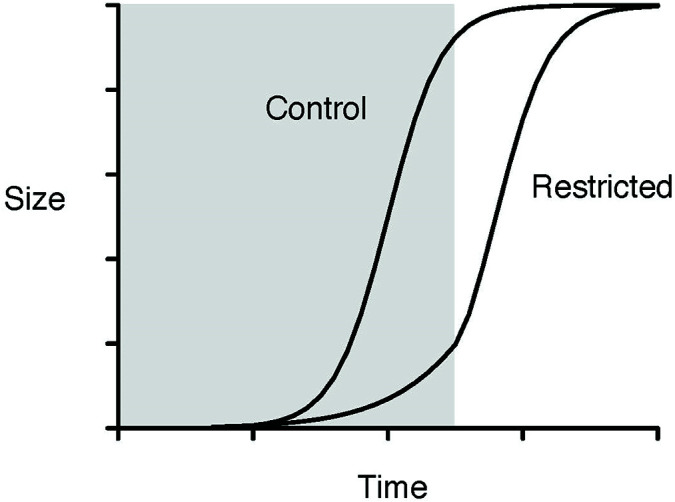
Hypothetical growth curves of control and diet-restricted treatment groups in the slope analysis. The shaded area represents the restriction period, and the non-shaded area represents the period after restriction (redrawn from Hector and Nakagawa (2012)).

Literature about CG contains some explanations for the CG phenomenon. One such hypothesis is the hypothetical growth curves of control and diet-restricted treatment groups, which represent the comparisons used in the slope analyses, where the slope of size increments in a treated group is higher than that from a control group. The situation of PCT, which is different from a normal completed CG process, is represented by the shaded area of **Figure 1** where PCT caused immediate biomass (size) removal and the effect of PCT to stand volume is readily apparent, and the CG process following PCT occurs right after the boundary between the shaded and non-shaded areas. The timing of thinning (PCT *vs.* CT) will determine the location of the boundary between the shaded and non-shaded areas, and the period required to reach full CG. Nevertheless, the final sizes of both groups are assumed to be the same, i.e., full CG.

This scenario can be explained in the mathematical form of

**Figure 2:**
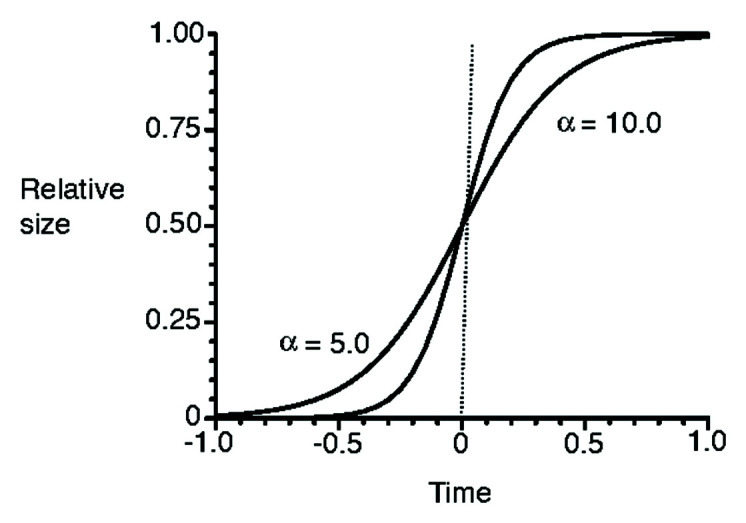
Sigmoid curves with two different values of *α* (redrawn from Onoda et al. (2011)).

(3) Size=K/(1+exp(α−β×Time))

where *K* is carrying capacity, and *α* and *β* are parameters. **Figure 2** shows the sigmoid curves with a different parameter *α*. A higher value of *α* could result in reaching the carrying capacity *K* earlier than with a lower value of *α*.

If this explanation holds, empirical evidence should be sought in the field of silvicultural research. A good example can be gleaned from our Shawnigan Lake PCT and fertilization experimental data analysis (Li et al., 2018), and this example could provide a starting point for identifying conditions of overcompensation.

### Shawnigan Lake PCT and Fertilization Trial Overview

**Figure 3:**
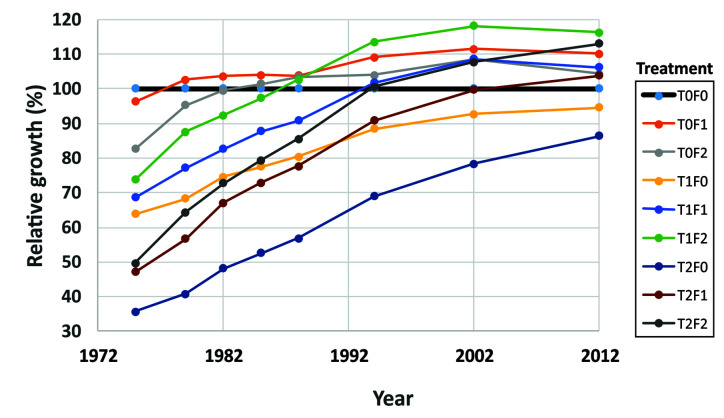
Relative growth of stand volume over time under different treatments (redrawn from Li et al. (2018)).

The Shawnigan Lake PCT and fertilization experiment provided a good example of how CG and overcompensation occurred over time, because of the multiple measurements taken before and 1, 3, 6, 9, 12, 15, 24, 30, and 40 years after the treatments. Each measurement was conducted at each of the combinations of three levels of PCT (0, 1/3, and 2/3 basal area removal) and three levels of fertilization (0, 224, and 448 kg N/ha). Each combination had either two or four replications (Crown and Brett, 1975). Consequently, the data analysis produced a detailed characterization of how forests responded to different treatments and clearly showed the processes of CG and overcompensation over the 40-year time horizon. The relative growth of stand volumes in each combination of PCT and fertilization levels to that from the control (T0F0) appears to be the best indicator of progressive changes of CG and reaching overcompensation (**Figure 3**).

The volume-based analytical results showed that the numbers of years to reach equality of volume was dependent on the levels of fertilization and thinning intensity. For example, if the thinning intensity was fixed at 50% basal area removal, reaching full CG could require less than 30 years when 448 kg N/ha was applied, about 35 years when 224 kg N/ha was applied, and over 60 years when no fertilizer was applied. A value-based assessment could display this trend with a clearer picture: lumber value can serve as an alternative indicator of CG (unpublished results, will be presented in Li et al. in prep.). The results confirmed that favoring lumber production and overcompensation could be reached with PCT operations, and the combination of PCT and fertilization treatments should be supported in forestry practices.

We can take a step further from these primary findings to see how they can be translated to an interpolation of stand growth trajectories under different thinning intensities over time for each level of fertilizer application. This can be done by fitting relative volume (percent of control) using the surface fitting program TableCurve 3D, and using the equation

(4) $$z=a+bx+cy+dx^{2}+ey^{2}+fxy$$

where *x* is percent of basal area removal or thinning intensity, *y* is year after thinning, *z* is relative stand volume expressed as percent of control sites, and *a*, *b*, *c*, *d*, *e*, and *f* are parameters with following values in **Table 1**:

**Table 1:** Parameters from a smooth surface fitting analysis.

Fertilization level	Parameter value					
	a	b	c	d	e	f
F0	95.82748	−0.90492	0.57782	−0.00330	−0.01314	0.02332
F1	90.13417	−0.79458	1.61649	0.00026	−0.02935	0.01884
F2	80.11890	0.03164	2.22308	−0.01263	−0.04210	0.02160

**Figure 4:**
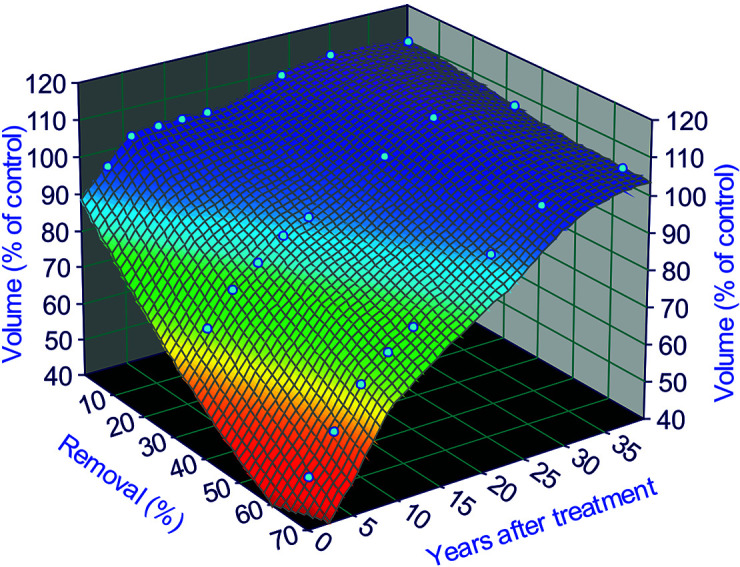
Surface fitting results for a fertilization level of 224 kg N/ha treatments in the Shawnigan Lake trial. This relationship can only be applied from year 1 to 40 after thinning treatments, because there are no data after 40 years and any extrapolation beyond this range might result in potential bias. This is due to the uncertainties involved in the growth trajectories after 40 years, and we will deal with these uncertainties in *Extrapolation of Growth Trajectories Beyond the Periods of Current Observations*.

**Figure 5:**
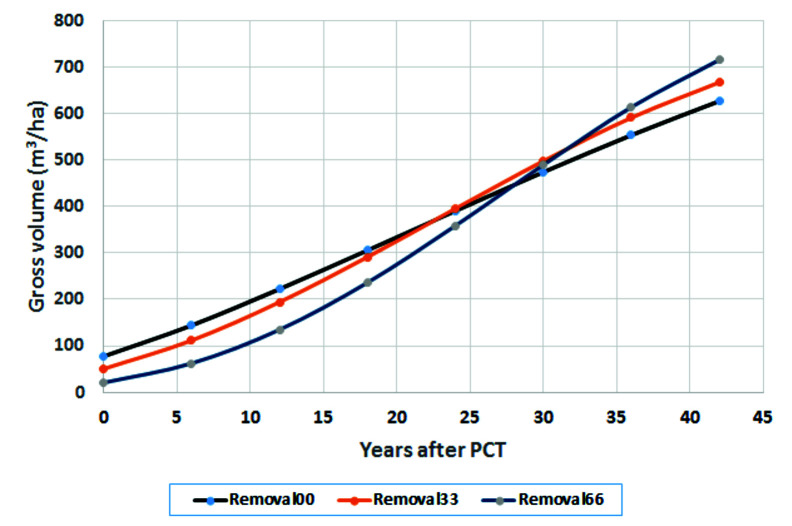
Growth trajectories after precommercial thinning (PCT) at the fertilization level of 224 kg N/ha.

To illustrate the fitting results, we use the fertilization level of F1 as an example (omit F0 and F2 for now), we have **Figure 4**:

For the current range of 1–40 years after treatments, we can multiply the normal growth trajectory (from control sites) by the *z* value to obtain altered growth trajectories. For example, applying the equation for the F1 level to the normal growth trajectory projected by using the Y-XENO model results in the growth trajectories in **Figure 5**; an economic evaluation of the Shawnigan Lake trial was carried out by Duke et al. (1989).

Removal00 means control (zero basal area removal), which is equivalent to normal growth trajectory. Removal33 and Removal66 (which represent 33 and 66% basal area removal, respectively) altered the slopes of the growth trajectories and resulted in probably reaching the status of stand maturity earlier. The figure clearly shows the shapes of altered growth trajectories by PCT treatments have a steeper slope in growth pattern than that in the normal/controlled sites. This is consistent with what Soucy et al. (2012) observed in long-term effects of thinning on growth and yield of an upland black spruce stand in Baie-Comeau, Quebec. The authors found that heavily thinned plots (50% of total basal area removal) resulted in a net stand merchantable volume increment 33% greater than that of the unthinned plots. In spite of a spruce budworm outbreak at the affected site, the heavily thinned plots maintained a superior tree growth rate and did not show senescence mortality like the other plots, allowing stand volume to catch up to that of the unthinned plots after 33 years. These provide a foundation for our scenario analysis of how future wood supply represented by regional annual allowable cut (AAC) could be affected by different shapes of growth trajectories (unpublished, to be presented elsewhere).

### Definition and Numerical Examples of Compensatory Growth

With the previously mentioned analysis, we can discuss further the definition of CG and explain it using numerical examples.

According to Tuomi et al. (1994), some plants can compensate, and even overcompensate, for the loss of productivity caused by herbivory. McNaughton (1983) and later Rea and Massicotte (2010) defined CG as “exaggerated vegetative growth that results from mechanical damage to plants (e.g., cutting, animal browsing, or breakage from snow) as a physiological consequence of an increase in the root-to-shoot ratio following the loss of aboveground biomass.” In general, CG has often been referred to as the process of accelerated growth of an organism following a disturbance or a period of slowed development due to unfavorable conditions such as low temperature or nutrient deprivation. In studies of fish behavior, as an example, CG has been referred to as a phase of accelerated growth when favorable conditions are restored after a period of growth depression (Ali et al., 2003).

**Figure 6:**
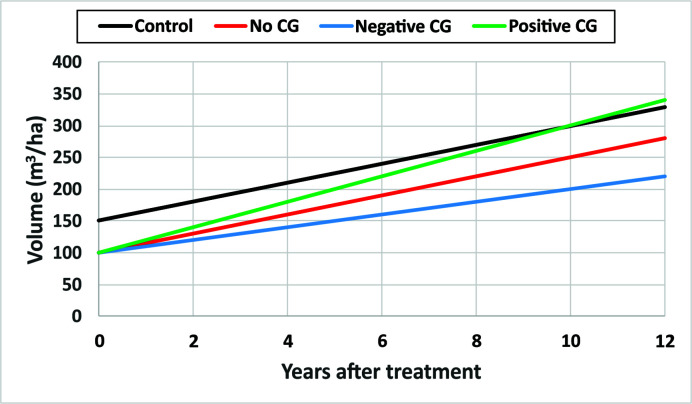
A schematic diagram of compensatory growth (CG) continuum with hypothetical numerical examples.

CG is not having only a single invariant state, but a continuum ranging from negative CG when mean periodic annual increments of DBH and/or *TopHt* in treated sites, *mPAI_Treat_*, is smaller than that from untreated sites, *mPAI_Control_*; to no CG when *mPAI_Treat_* equals *mPAI_Control_*; and to positive CG when *mPAI_Treat_* is bigger than *mPAI_Control_*. Here the *mPAI* is calculated as $mPAI_{i}=(Vol_{i,t_{2}}-Vol_{i,t_{1}})/(t_{2}-t_{1})$, where *i = Treated*, or *Control*, *t*_1_ and *t*_2_ are the beginning and end time of the observation period, respectively. **Figure 6** is a schematic diagram of CG continuum.

Some hypothetical numerical examples are used to demonstrate different types of CG for narrative description purposes. Assuming stand volumes in an untreated site are 150 and 300 m^3^/ha at year 0 and year 10 after the initial treatments as showed in black line, the *mPAI_Control_* will be 15 m^3^/ha. If the thinning operation removed 1/3 of stand volume in the treated site and the stand volume, measured 10 years after the initial treatment, is 250 m^3^/ha as shown in red line, the *mPAI_Treat_* will also be 15 m^3^/ha. In this case, there is no CG observed since *mPAI_Treat_* = *mPAI_Control_*. However, if the stand volume measured 10 years after the initial treatment is smaller than 250 m^3^/ha, say 200 m^3^/ha as showed in blue line, the *mPAI_Treat_* will be 10 m^3^/ha, and it will then be a negative CG since *mPAI_Treat_* < *mPAI_Control_*. If the stand volume measured 10 years after the initial treatment is larger than 250 m^3^/ha, say 300 m^3^/ha as showed in green line, the *mPAI_Treat_* will be 20 m^3^/ha, and it will be a positive CG since *mPAI_Treat_* > *mPAI_Control_*.

The negative CG, which causes thinning shock—an unexpected result of reduction in diameter and height growth—is probably rare and short-lived, and would then shift to no CG or positive CG after that period. Harrington and Reukema (1983) provided an example of negative CG observed in a spacing experiment through thinning on a Douglas-fir plantation in the Wind River Experimental Forest near Carson, Washington. The PCT treatments were designed for six stocking levels 875, 625, 500, 375, 250, and 125 trees per hectare (corresponding to spacings of 3.4, 4.0, 4.2, 5.0, 6.0, and 8.1 m) with a randomized block design. They found the immediate effects of the thinning treatments were detrimental including chlorotic foliage, sunscald, and snow and ice damage in the form of broken tops and branches and leaning trees. The height and diameter growth were reduced by thinning. However, the long-term (15–25 years after treatments) effects of increased growing space have been beneficial. Following the initial shock, both diameter and height growth did recover in the thinned plots. In fact, diameter and height growth are greatest at the widest spacing. Nevertheless, due to the significantly fewer trees per plot at the wide spacings, the sharp increase of basal area and cubic volume per hectare have not been great enough to offset the lesser number of trees.

Alternative explanations for the negative CG could simply mean that the organisms are constrained from catching up, for example, if trees performed better in dense groups wherein they reduce herbivory. Or there could be some limiting nutrient (e.g., N) that prevents the tree population from exceeding growth rates of non-thinned trees *via* CG. However, these phenomena will not be the focus of this current synthesis.

The example presented by Harrington and Reukema (1983) clearly showed how a negative CG translated into positive CG *via* no CG over the 25 years of observations, in which the no CG state was temporary. This has been theoretically confirmed by Jaremo and Palmqvist (2001). They presented a theoretical analysis that considers the phenotypic trait of CG ability in a context of population dynamics. Their model depicts a system of three interactors: herbivores and two diﬀerent plant types referred to as ordinary and compensating. The compensating plant type has the ability to increase its intrinsic rate of biomass growth as a response to damage. This CG ability is maintained at the expense of a reduced growth rate in the absence of damage, where the ordinary plant type has the higher growth rate. Analysis of this system suggested that, even though a compensatory capacity of this kind will not imply an increase in equilibrium plant density, it will give a competitive advantage in relation to other plants in the presence of a suﬃciently eﬃcient herbivore. Invasion of compensating plants into a population of non-compensating plants is facilitated by a high CG ability and a high intrinsic rate of plant biomass increase. Conversely, an ordinary plant can invade and outcompete a compensating plant when the herbivore is characterized by a relatively low attack rate, and/or when the intrinsic growth rate of a plant is decreased.

The status of positive CG can be further distinguished as undercompensation, compensation, and overcompensation. To evaluate the exact status of positive CG, we can extrapolate the lines beyond the interception of the two straight lines. The location of the intercept indicates where the compensation happens. Before reaching the intercept, there is an indication of undercompensation, and after the interception denotes an overcompensation status, which is what the forestry practitioners seek. In the case presented by Harrington and Reukema (1983), however, overcompensation has not occurred during their 25-year observation horizon despite a transition from negative to positive CG.

Behavioral ecology views CG as a process essentially at the individual tree level. This is because natural selection operates on individuals in a population and selectively favors those individuals and those genotypes that contribute most offspring to subsequent generations. This process leads continuously to an increase in the population of those forms that contribute more offspring than their neighbors. We define “fitness” as the relative numbers of offspring left to future generations by one form compared with others. For this reason a plant might increase its fitness by reducing its number of progeny if a compensating increase in its vegetative vigor deprived its neighbors of the chance of leaving offspring (Harper, 1977). In other words, reduction of the plant numbers in a given area (e.g., a result from thinning) might not necessarily lead to the decrease of fitness, because they could potentially be compensated by increased individual plant sizes that could potentially result in more offspring compared with those from unthinned stands.

Thinning operations may alter the status of environmental conditions for surviving trees, such as reduced competitive pressure and increased availability of resources and nutrients. As a result, individual trees can devote more resources to growth because fewer reserves are required to compete with neighbors. This could be a mechanism of released growth after thinning. Nevertheless, this natural selection favored an individual-level CG process, which could eventually lead to an expression of CG at the population level, if such growth persists for a sufficient period of time.

In **Figure 6**, the status of positive CG can be demonstrated when the positive (green) and control (black) lines are extended further such as at 12 years. The partial (or insufficient) CG (or undercompensation) refers to the status of stand volumes in treated sites that are in the process of catching up with the one from untreated sites, represented as the portion of positive (green) line 1–9 years after initial treatment. In other words, the difference between stand volumes in treated and untreated sites is narrowing over time. The full CG, or compensation, denotes that no significant difference between the stand volumes in treated and untreated sites, represented as the year 10 after initial treatment where the interception of lines that represent control (black) and no CG (red) lines occur. This compensation can also be called compensation-induced equality (CIE). Overcompensation signifies that the stand volumes of treated sites exceed the one from untreated sites, represented by the portion of positive (green) line in 11–12 years after the initial treatment.

The above method can be applied to every interval between two measurements in the datasets containing multiple measurements. Resulting *mPAI_Treat_* and *mPAI_Control_* may or may not be the same over time, indicating the speed of CG process may not be invariant and could change over time.

It should be noted that the numerical examples in **Figure 6** are for illustrating the CG related concepts only, and they are not necessarily realistic. In reality, growth rates of trees are usually much slower than the numerical examples used in **Figure 6**, which is probably the reason why the catch-up growth was not observed in short-term observations.

The framework of CG can reasonably explain the different results from short- and long-term observations, and indicate that released growth at the individual tree level is a necessary but not sufficient condition for stand volume catch up, and the sufficient condition is a time period long enough to allow the volume catch up to occur (Li et al., 2018). An advantage of the concept of CG is to allow researchers to focus on the positive CG process for potential applications in forestry practice. The CG concept is thus employed in the current study to explore and quantify how the normal growth trajectories, denoted by the untreated sites, could be altered by the CG process induced by the combinations of PCT and fertilization treatments at the Shawnigan Lake study area.

## Possible Generalization of Overcompensation to Other Species and Geographical Regions

Though the concept of overcompensation observed at the Shawnigan Lake trial is important in relation to forest productivity enhancement and carbon accounting, two challenges remain: how to extrapolate growth trajectories beyond the periods of current observations and how to generalize overcompensation to other tree species and geographical regions.

### Extrapolation of Growth Trajectories Beyond the Periods of Current Observations

**Figure 7:**
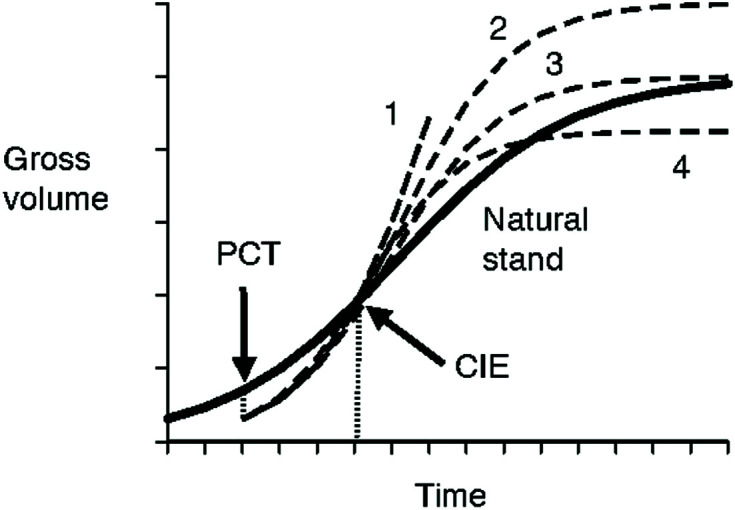
Schematic illustration of uncertainties in long-term growth trajectories. The bold line indicates growth trajectory of natural stands. The gross volume will be reduced at the precommercial thinning (PCT) treatment, then gradually approach that of the natural stand growth curve within a certain period of time at the point of compensatory-induced-equality (CIE), and then face uncertainties with possible growth trajectories indicated by 1 (continued growth acceleration); 2 (higher maximum growth); 3 (no change to maximum growth); and 4 (lower maximum growth).

The issue with the previously identified first challenge (how to extrapolate trajectories beyond the periods of current observations) lies in the uncertainties involved with growth trajectories of even longer periods of time (i.e., data from the Shawnigan Lake trial were only available for up to 40 years after the initial treatments) that have been mentioned in *Shawnigan Lake PCT and Fertilization Trial Overview*. The uncertainty could at the very least include following possibilities (**Figure 7**):

- forest productivity continues its growth increment over time due to accelerated growth rate (Huuskonen and Hynynen, 2006);
- forest productivity flattens at the same level as observed in natural stands, which is assumed to be the maximum site productivity;
- forest productivity flattens at a level higher than the observed productivity in natural stands, wherein potential maximum site productivity is higher than what has been observed in natural stands; and
- forest productivity declines gradually to a level lower than that observed in natural stands.

To deal with such uncertainties, re-measurements will be required in the years to come when taking an empirical data collection approach. For example, the Shawnigan Lake trial was initiated in 1971 and the last measurement was made in 2012, which consisted of 40 years of records. When future re-measurement becomes possible, the collected data could be used to provide an adequate answer to the uncertainty. For the Green River case, re-measuring some of the study sites is impossible because the trees have been felled, but re-measuring nearby sites with similar treatments could still provide useful information to resolve the uncertainties. The modeling approach is an alternative option, which will be discussed later in *Plant Response Manipulation Through Optimal Stimuli/Thinning Determination*.

### Generalization Using CG for Other Tree Species and Geographical Regions

The other previously identified issue is to what extent the CG and overcompensation observation from a single site (Shawnigan Lake trial) could be generalized for other tree species and geographical regions. Two different approaches are available to explore the issue: empirical data collection and further analysis as well as modeling approach.

#### Hypothesis Testing Through Increased Empirical Data Collection and Analysis

Empirical data collection and analysis largely relies on analysis of accumulated empirical data from existing sites of long-term PCT trials for other tree species and from different geographical regions. If the hypothesis for the existence of overcompensation can be supported from these data analyses, one can comfortably generalize the overcompensation using other tree species and geographical regions.

This approach appears to be intuitively acceptable, relatively straightforward, reasonable and understandable, and easily convincible. However, this approach is also very resource demanding in terms of both time and economic investment in a skilled workforce. Furthermore, suitable potential sites for long-term (at least 40–50 years) PCT trials need to be identified that are readily available and accessible.

Considering the long history of silviculture research, suitable potential sites could be easily identified as long as establishment measurements (before and after treatments) are available. For example, the following sites in western Canada could serve as potential sites:

- Historical thinning treatments (approximately 50 years ago) established for the coastal Douglas-fir in the Malcolm Knapp Research Forest of UBC in Maple Ridge, B.C.;
- Diverse thinning experiments 40–50 years ago launched in the mixedwood forests near Calling Lake in Northern Alberta;
- Various PCT experiments initiated in Manitoba, Canada, more than 70 years ago.

Even with sufficient financial investments, however, there might still be the possibility of insufficient suitable sites for all major tree species in all geographical regions. When this is the case, other thinning experiments need to be set up for data collection in subsequent years. These experiments will require a sufficiently long period of observations to be appropriate for the hypothesis test. Obviously, this will probably be a task to be accomplished beyond any given researcher’s career limit.

With the previously described empirical data collection, the results still might be treated as anomalous or out of the ordinary due to the lack of explanatory mechanisms. Technically, this approach could still face challenges in implementation:

- Human errors or biases in the measurements spread over a long period by different researchers, with possible varying research protocols that might complicate comparative data analysis;
- Inconsistent methods of data analysis over a long period could also complicate research results. For example, different methods of estimating volumes of individual trees over the 40-year horizon resulted in significant differences in stand volumes published previously using the Shawnigan Lake trial datasets, hence infeasible data unsuitable for direct comparison;
- Experiment duration challenges include long time scale (probably more than 50 years) and uncertainties related to selection of variables to measure. In this regard, a modeling approach could probably benefit researchers through the use of simulations and a sensitivity analysis (see *Plant Response Manipulation Through Optimal Stimuli/Thinning Determination*).

#### Plant Response Manipulation Through Optimal Stimuli/Thinning Determination

The modeling approach proposed here takes advantage of the contemporary development from other research fields, especially from applied behavioral ecology, which has been successfully applied in other management science such as pest management in agriculture.

Different methods including chemical, physical, and biological controls have been traditionally practiced in pest management for protecting normal growth of plants. A new method of behavioral manipulation was recently added to the list (Roitberg, 2007). This new method is a result from applied behavioral ecology with the advantage of minimized environmental footprint.

In the study of thinning effect on forest productivity, thinning acts as a stimulus to forests, and how forests respond to such stimuli (i.e., forest behavior) is represented as CG. The goal of thinning operations in forestry practices is to obtain maximized forest productivity, such as the overcompensation observed in the Shawnigan Lake trial. Thus, the focus of research should be determining the optimal stimuli that can result in the maximized level of overcompensation. Such a research goal is similar to behavioral manipulation in pest management wherein optimal stimuli are supplied to reduce damage by pests or increase efficacy of natural enemies of pests. Therefore, we propose the following new state-dependent forest growth model, which is based on principles from behavioral ecology.

Without any disturbances, the stand variables could be projected according to normal growth trajectories, similar to other simulation models of stand dynamics. With a disturbance, however, the growth trajectories will be altered according to the nature of the disturbance. Huuskonen and Hynynen (2006) developed an analytic model with a logarithm form to predict diameter increment as a function of dominant height, initial stand density and the actual stem number of the growing stock, and the regeneration method. We propose that thinning (an anthropogenic disturbance) can be characterized by three components: timing, intensity, and method. In other words, any thinning regime could be expressed as a combination of these three variables, with a given forest responding to the specific variables. Here, the key issue is how a forest might respond to disturbance, and we propose that the response would be based on the state of tree (e.g., age, size, and energy reserve) and the level of stimuli (thinning). According to the first principle of evolutionary biology, the best response should have evolved over past millennia, with maximized fitness that is favored by natural selection. If this is reasonable, one should be able to calculate the best response accordingly (Smith, 1978; Parker and Smith, 1990).

Behavioral ecologists have developed an algorithm for such a calculation. For example, to investigate masting “behavior” of trees, Lalonde and Roitberg (1992) developed a method of calculating a decision matrix representing the best behavioral response to the environment under various conditions. Their conceptual framework is described below:

Most plants acquire resources as a result of photosynthesis. The rate of resource acquisition is a function of the quantity of resources previously allocated to the vegetative growth including the creation and maintenance of photosynthetic structures (e.g., leaves), support structures (lignified tissues), and uptake structures (roots). In order to accrue fitness, a plant must, at some point, divert some of its resources away from vegetative growth and toward reproduction (seeds and seed support structures). The cost of reproduction in terms of depressed capacity or vegetative investment during fruiting years is well demonstrated for masting plants, and a plant must, therefore, balance its allocation to vegetative versus reproductive investment over its life time in order to maximize its reproductive success. This is essentially the stuff of life history theory that takes explicit recognition of state-dependent costs and benefits into account (see *State-Dependent Forest Growth Models* on state dependence). Lalonde and Roitberg thus developed a stochastic dynamic optimization model to determine how the dynamics of resource acquisition can lead to fluctuations in seed production in a long-lived perennial. The stochastics are based upon environmental inputs (e.g., light, water, nutrients) and dynamics refer to changes in values of state variables (e.g., roots and shoots) associated with plant response. The optimization equation takes the form of

(5) $$\begin{array}{l} F(V,S,t,T)=\max\{P_{g}F(V-C\times V+Q_{g},S+(R_{g}-R_{g}D),t-1,T)\\ +(1-P_{g})/2\times F(V-C\times V+Q_{f},S+(R_{f}-R_{f}D),t-1,T)\\ +(1-P_{g})/2\times F(V-C\times V+Q_{b},S+(R_{b}-R_{b}D),t-1,T)\} \end{array}$$

where *V* is vegetative growth; *S* is the number of seeds that it has already produced; *t* is current age in relation to its maximum life span *T*; *P_g_* the probability of experiencing a good growth year; *C* is the maintenance factor; *D* is a start-up cost; *Q* and *S* are resources allocated to vegetative and reproductive growth, respectively; *R* is current investment in seed production; and the subscript’s of *g*, *f*, and *b* indicate good, fair, and bad growth year, respectively. This equation was solved at (*t=T*) for all states of vegetative and reproductive investment and then iterated backward in time (i.e., backward induction) for a 100-year life span. The results were used to construct a decision matrix that holds the calculated optimal investment level to vegetative growth and to reproduction for all possible states and times (i.e., the matrix provides an analytical solution to the problem of living in a stochastic world with known probabilities of events and related outcomes).

After the decision array was constructed, Lalonde and Roitberg (1992) were able to investigate the effect of an optimized life-history strategy on the emergent properties of the seed production population, using a stochastic simulation model. Without any further details, this outlined simulation procedure provides a new approach of developing a state-dependent forest growth model where anthropogenic disturbance (e.g. PCT) is explicitly included to predict growth response of surviving trees in a stand.

The advantage of this new modeling approach is its capability of analytically answering some key questions about the generalization of CG to other tree species and geographical regions. With parameters specific to a tree species of concern, this new model is expected to produce growth trajectories under given management scenarios such as different thinning regimes.

## Overcompensation and Wood Supply Shortage

Through vast forest areas across Canada, unfavorable climate and extreme weather conditions have caused most trees to grow slower than in many other parts of the world. Despite expectations of better-quality wood fiber resulting from slow-growing trees in general, mean annual increment (MAI) also may be lower, which would inevitably lead to insufficient wood supply for the increasing demands from markets. Consequently, balance between demands of utilization and concerns of sustainability and environment protection have become important in fulfilling goals of sustainable forest management.

Overcompensation provides enhanced forest productivity, which would have a profound effect on current and future wood supply. As a result, searching for conditions for promoting overcompensation could form a new mitigation strategy for dealing with the issues related to wood supply shortage. In other words, overcompensation adds a new pillar to the existing strategies of forest productivity:

- Borrowing some future wood supply to satisfy market opportunities: adjust harvest activities to allow fluctuation according to market demand. This appeared a good strategy from a market economic perspective; however, it could create unfavorable conditions for mill operations;
- Relaxing current harvest constraints: harvest activities would have increased flexibility and thus lead to temporal fluctuations of wood supply;
- Improving efficiency of wood utilization: this can be achieved mainly by two means: (a) produce more log volume from the same forests, such as making changes in wood utilization standards (e.g., Li et al., 2016a), produce more products without increasing the total volume of harvested wood such as optimization of sawing technology (Li et al., 2016b).
- Developing new managed forest areas: expansion of existing managed forest area through building infrastructures for accessibility and markets, this needs to be taken into account at higher levels of planning and during decision-making processes.

This new mitigation strategy differs from the above four existing strategies because of the context of enhancing forest productivity, instead of only being based on current forest productivity. This strategy aims at increasing forest productivity through overcompensation and earlier than normal achievement of stand maturity, and hence elevating regional annual allowable cut that is essentially based on the long-term average forest productivity. Such a proactive mitigation strategy could provide an environment that satisfies industrial demands without compromising the goals of sustainable forest management.

## Concluding Remarks and Recommendations

CG is common in organisms from both plant and animal kingdoms despite different terminologies used to describe them in the literature. CG encompasses a continuum from undercompensation to full compensation, and to overcompensation. Overcompensation is of particular interests in the field of forestry because it results in enhanced forest productivity. Studying the overcompensation mechanisms is important because it could reveal whether the Shawnigan Lake case and Green River thinning trials could be generalized to other tree species and geographical regions, and thus benefit the forest sector. Searching for and creating conditions for promoting overcompensation could form a new mitigation strategy for dealing with issues related to wood supply shortage. Results from such studies could provide cost-effective decision support tools to forestry practitioners.

To accomplish this new mitigation strategy, we recommend to conduct several tasks: (1) further clarification of CG concept to avoid misunderstanding and misapplication in forestry practice; (2) identification of alternative indicators of CG that are compatible with forestry practice; (3) collection of empirical datasets from legacy silviculture sites; and (4) development of flexible and user-friendly tools for predicting CG for given stand types and site conditions. These tools should be state-dependent, and combined using optimization and simulation technology, an approach developed in behavioral ecology, can determine the conditions under which overcompensation could occur and then refine the model by incorporating species-specific parameters for predicting whether and how overcompensation could be expected for a region under management.

## Data Availability Statement

The data analyzed in this study is subject to the following licenses/restrictions: Datasets used in this review and synthesis paper were published in the literature. Requests to access these datasets should be directed to chao.li@canada.ca.

## Author Contributions

CL, HB, BR, and RL jointly conceived and wrote the paper.

## Funding

This work was financially supported by Natural Resources Canada-Canadian Forest Service’s Developing Sustainable Fibre Solutions Research Program.

## Conflict of Interest

The authors declare that the research was conducted in the absence of any commercial or financial relationships that could be construed as a potential conflict of interest.
